# Intelligent Urban Public Transportation for Accessibility Dedicated to People with Disabilities

**DOI:** 10.3390/s120810678

**Published:** 2012-08-06

**Authors:** Haiying Zhou, Kun-Mean Hou, Decheng Zuo, Jian Li

**Affiliations:** 1 School of Computer Science, Harbin Institute of Technology, Harbin 150001, China; E-Mails: haiyingzhou@hit.edu.cn (H.Z.); lijian_susan@hit.edu.cn (J.L.); 2 LIMOS Laboratory, University of Clermont-Ferrand II, Clermont-Ferrand 63000, France; E-Mail: kun-mean.hou@isima.fr

**Keywords:** public urban transportation service access, people with disabilities, urban environmental surveillance, fault-tolerant component-based architecture, resource awareness communication and scheduling

## Abstract

The traditional urban public transport system generally cannot provide an effective access service for people with disabilities, especially for disabled, wheelchair and blind (DWB) passengers. In this paper, based on advanced information & communication technologies (ICT) and green technologies (GT) concepts, a dedicated public urban transportation service access system named Mobi+ has been introduced, which facilitates the mobility of DWB passengers. The Mobi+ project consists of three subsystems: a wireless communication subsystem, which provides the data exchange and network connection services between buses and stations in the complex urban environments; the bus subsystem, which provides the DWB class detection & bus arrival notification services; and the station subsystem, which implements the urban environmental surveillance & bus auxiliary access services. The Mobi+ card that supports multi-microcontroller multi-transceiver adopts the fault-tolerant component-based hardware architecture, in which the dedicated embedded system software, *i.e.*, operating system micro-kernel and wireless protocol, has been integrated. The dedicated Mobi+ embedded system provides the fault-tolerant resource awareness communication and scheduling mechanism to ensure the reliability in data exchange and service provision. At present, the Mobi+ system has been implemented on the buses and stations of line ‘2’ in the city of Clermont-Ferrand (France). The experiential results show that, on one hand the Mobi+ prototype system reaches the design expectations and provides an effective urban bus access service for people with disabilities; on the other hand the Mobi+ system is easily to deploy in the buses and at bus stations thanks to its low energy consumption and small form factor.

## 1. Introduction

Traditional urban public transportation systems worldwide are generally designed for a healthy population and rarely take into account the needs of people with disabilities. The United Nations estimates that between 6 and 10% of the population in developing countries and some 400 million people worldwide have a disability [1]. Moreover, the number of persons with disabilities will increase significantly in the next decade due to the increasing number of elderly people. In [2], NTIS surveys the activities and progresses in America's transportation systems and services for people with disabilities in the United States since 1990. Baudoin *et al.* [3] revealed the progresses of advanced transportation systems for disabled people in France. Mashiri *et al.* [4] reviewed the status of public transportation services for persons with disabilities in the developing world. All the survey results show that in order to improve the quality of life for people with disabilities, both developed countries and developing countries need to improve the accessibility of the urban public transportation and to make it more attractive. We note that on the one hand transport is an important enabler of strategies to fight poverty through enhancing access to education, employment, and social services [5] and on the other hand, it is a key issue to reduce traffic jams (air pollution) in big cities. Moreover the urban public transportation accessibilities for people with disabilities are important factors in reducing poverty and can facilitate the participation of people with disabilities in economic, social and political processes [1].

In order to improve the accessibility of urban public transportation system for the people with disabilities different domains must be investigated such as social science (user requirements and acceptation, economic and social impacts, sustainable development …), information (real-time urban transportation traffic, traffic optimization and simulation flow, urban air quality …) and infrastructure (security, quality of service for accessibility and comfortable of waiting room at bus stops or at multimodal stations …) and vehicles (comfortable dedicated seats or spaces for disabled people (e.g., wheelchair users), low-floor buses for accessibility, *etc.*). Some studies focus on modeling the travel behaviors of people with different kinds of disabilities and then giving the service provision suggestions for traditional urban public transportation systems [6,7]. Some dedicated public transportation systems for people with disabilities are thus implemented [3,4,8].

This paper is organized as follows: Section 2 describes the accessibilities (services) for people with disabilities provided by the Mobi+ project, Section 3 illustrates the dedicated embedded system of the Mobi+ card in details, including the hardware structure, the operating system micro-kernel and a dedicated application protocol, Section 4 gives a functional description of the Mobi+ system; in Section 5, we draw conclusions and present the ongoing work.

## 2. Accessibilities for People with Disabilities

Currently, in spite of the important advances in transportation systems in developed countries (e.g., European Union) the accessibility to the urban public transportation system still does not meet the requirements of people with disabilities. To face the increasing numbers of people with disabilities in the next decade, the Syndicat Mixte de Transport en Commun de l'agglomération clermontoise (SMTC) transportation union of Clermont-Ferrand (France) launched the Mobi+ project [9], which aims to improve the accessibility (services) to urban public transportation to meet the requirements of Disabled, Wheelchair and Blind (DWB) people by adopting advanced information & communication technologies (ICT) and green technologies (GT) concepts. In order to facilitate the accessibility to urban public transportation for people with disabilities different improvements must be carried out jointly to vehicles (buses, tramways, trains, subways, *etc.*), infrastructure and information.

One of the issues to facilitate bus access for people with disabilities is that the bus floor must be level with the pavement and the distance between the pavement and the bus floor must be less than 5 cm (the EU standard). If these conditions are achieved, persons with disabilities can get in/out the bus autonomously. If the height of the pavement is not the same as the bus floor one, three solutions may be adopted: (i) rebuild the pavement (economically unacceptable in old cities); (ii) dynamically adapt bus floors to the height of the pavement; (iii) deploy pallets. The last two solutions need extra time. In the Mobi+ project, the third solution is adopted to minimize the cost by using the current available buses and retaining the old pavement.

In fact, to facilitate bus access for DWB passengers, the bus driver has to carefully park the bus close to the pavement (<5 cm) and to deploy the pallet at each bus station. This takes three minutes more than usual bus parking for healthy passengers but in general at the bus station the DWB passengers are not present. Thus for a bus line having 30 bus stops, 90 more min are needed if the bus driver has to park carefully and deploy the pallet for DWB passengers at every bus stop. Consequently for the same bus line to provide the same service (e.g., the same bus frequency), the number of buses must be increased significantly.

In order to improve the accessibility to the urban public transportation system for people with disabilities, it is important to signal to the bus driver the presence of the disabled people before the next bus stop. Hence an urban public transportation auxiliary access system dedicated to people with disabilities was implemented. In this system, the disabled people, including the passengers with baby buggies will take a specific ticket (RFID tag) to indicate the type of their handicap (e.g., wheelchair user). With the RFID tag, when the tag users arrive and wait at the bus station, their presence will be automatically detected and the bus driver will be thus informed, so that the driver will perform the bus parking carefully in order to correctly deploy the pallet.

Moreover, currently the air pollution in big cities attains a critical threshold for fragile passengers, that is why it is important to quantify in real-time the air quality of the city by embedding CO and NO gas sensors in the bus. So by combining the real-time gas sensors data of the buses with the fixed air pollution detection station ones, the quality of city air pollution may be quantified more precisely over the whole city.

**Figure 1.**
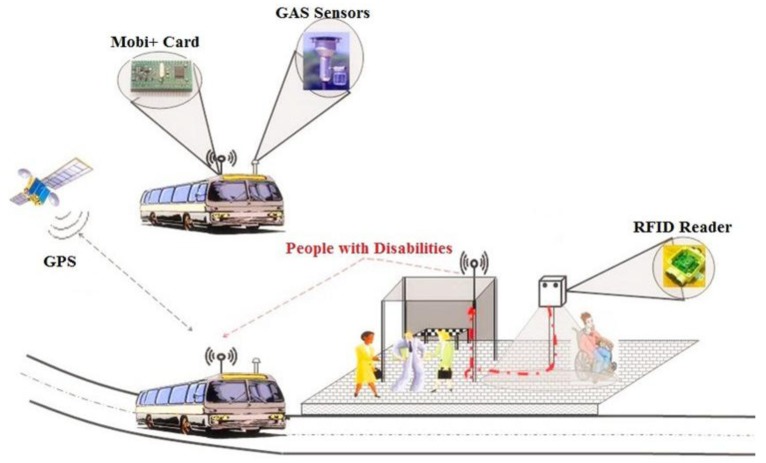
Functional bloc diagram of the Mobi+ system.

Our work is to implement an urban public transportation service access & urban environment monitoring system dedicated to people with disabilities with the features of robustness, low cost, small form factor and easy to deploy effectively, which is named Mobi+. In this paper we will focus on the research and implementation of Mobi+ system architecture. The system supports a reliable bus-station interaction in the complex urban environments and provides the following services: DWB class detection/alarm notification services in the station peer and environmental surveillance/bus parking & access services in the bus peer. Figure 1 shows the functional bloc diagram of the Mobi+ system.

## 3. Embedded System Architecture of Mobi+ Card

The core components of this Mobi+ system, termed Mobi+ card, are installed at bus stations and in buses respectively, being responsible for data exchange and service provisions. Based on the application-related design principle, the hardware and software architectures of the Mobi+ card are elaborated as follows.

### 3.1. Fault-Tolerant Component-Based Hardware Architecture

**Figure 2.**
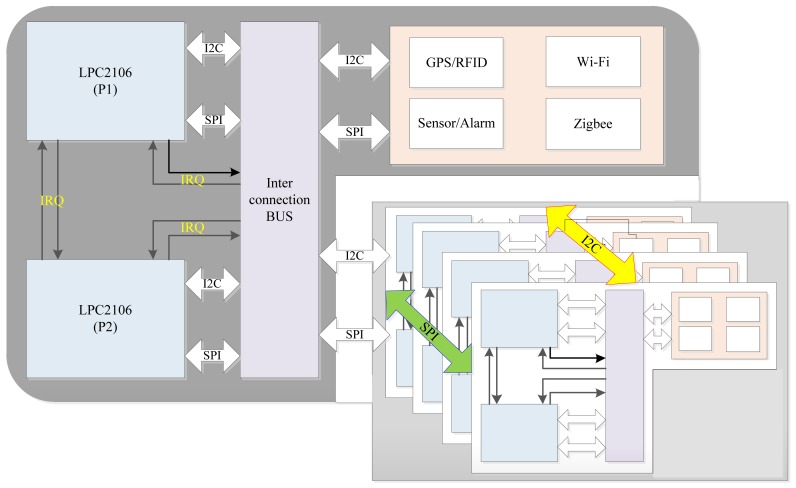
Fault-tolerant component-based architecture of Mobi+ cards.

The Mobi+ card implements a fault-tolerant component-based hardware architecture based on a multi-microcontroller multi-transceiver, as shown in Figure 2. There are two types of Mobi+ cards: the bus card that consists of a positioning unit (GPS), an urban environmental monitoring unit (*i.e.*, air-quality sensors, temperature sensor, *etc.*) and a bus service provision unit; the station card that has an identification detection unit (RFID reader) and an arrival alarm notification unit (*i.e.*, LED, Buzzer, Speaker, *etc.*). The Mobi+ card is characterized by the following features:

#### Multi-microcontroller architecture

In order to improve the system fault-tolerant capability, the Mobi+ card is equipped with two microcontroller chips, referred to as P1 and P2. In normal mode, P1 runs in operation mode to perform the system tasks, and P2 is configured to run in standby or sleep mode. In standby mode, P2 monitors the system operating situation based on the specific heartbeat detection logics. When a fault or abnormity is detected, two possible solutions will be performed according to the error type: (a) reset the Mobi+ card if the fault recovery is possible in P1; or (b) replace P1 by P2 to execute the system tasks in P2. It should be noted that P2 can be set to run in sleep mode to decrease the energy consumption. In the abnormal mode, P1 and P2 are set to work collaboratively to perform the same tasks so as to ensure the reliable data exchange and service provisions. In the Mobi+ card, P1 and P2 communicate with each other via SPI/I^2^C bus interface while P1 is the master and P2 is the slave by default.

#### Multi-transceiver support

In order to apply it in the complex and time-variant urban wireless communication environment, two different wireless access media (*i.e.*, WiFi and ZigBee) are adopted simultaneously in the Mobi+ cards to ensure a reliable data transmission between buses and stations through the low-quality wireless channel. At least two wireless devices (three devices in the station card) are implemented in the Mobi+ card. All the wireless devices in Mobi+ cards operate in the same 2.4 GHz Industry, Science and Medicine (ISM) frequency band, which may result in mutual frequency interferences between different wireless devices. In this system, we assign channel 1 for WiFi module (2.4120 GHz), channel 8 for RFID module (2.4537 GHz) and channel 13 for ZigBee module (2.4700 GHz). Experimental results demonstrate that this configuration guarantees the optimal communication performance.

#### Multi-alarm notification & multi-sensor surveillance

In the station peer, several alarm notification measures are employed to inform the DWB passengers of the arrival of buses: the RFID module for identifying the DWB classes; different blinking frequencies of the luminous indicator or alarms of the buzzer to inform the arrival of coming buses. In the bus peer, several different kinds of sensors are adopted to monitor the urban environments: the GPS module for localizing the bus position in the city; the air-quality sensors and the temperature sensor for collecting the urban environmental data at different times of a day.

### 3.2. Fault-Tolerant Resource Awareness Software System

The software system of the Mobi+ card is developed to meet the requirements of low-resource consumption and fault-tolerant capability. For this purpose, the specific choice of operating system and design of protocol stack are made.

#### A. Hybrid Embedded Real-time Operating System Micro-kernel: HEROS

The Mobi+ system adopts a lightweight hybrid operating system microkernel named HEROS [10] which integrates the advantages of multi-threading system (SDREAM) [11] and event-driven system (TinyOS) [12]. HEROS adopts the component-based system architecture, which can be configured to run in real-time the multi-threading mode or the event-driven mode to meet the requirements of practical applications.

- *Component-based hierarchy architecture*: HEROS contains two kinds of system components: *thread* and *event*. A thread is a functional component that performs a specified action and an event is the task component that indicates a specified behavior. An event can be considered as a package widget that encapsulates a group of threads to complete a task. Events are executed sequentially according to the event priorities. Events are interruptible but not pre-emptive. Threads belong to one event, and are performed concurrently according to the thread priorities. Threads are both interruptible and pre-emptive.
- *Tuple-based communication & synchronization mechanism*: Based upon the parallel concept of *LINDA* language [13], HEROS implements a *tuple* space and *IN & OUT* primitives for data exchange and internal communication among system components such as IO peripherals and microcontrollers. The *tuple* space that consists of a set of *tuples* provides a group of shared buffers for data exchange or signals management between components. The *IN & OUT* primitives implement *read & write* operations in the *tuple* space for system communication & synchronization.

To define a HEROS application ‘*H*’ having *n* independent events ‘*E*’ and an event *E_i_* is a logical encapsulation of *m_i_* related threads ‘*T*’. The symbol ‘ →’ indicates the sequential operation and the symbol ‘//’ represents the concurrent or parallelism operation, then:

$$\begin{array}{l} H=\{E_{i}:i=1,\ldots ,n,E_{1}\to E_{2}\to \ldots \;\ldots \to E_{n}\};\\ E_{i}=\{T_{ij}:j=1,\ldots ,m_{i,}T_{i1}//T_{i2}//\ldots \;\ldots //T_{\text{imi}}\}; \end{array}$$

**Figure 3.**
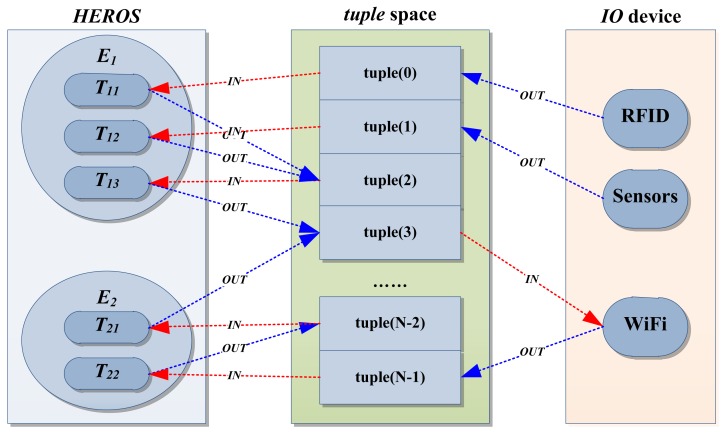
Schematic diagram of HEROS.

When *n* = *1, H* = *E_1_* = {*T_11_* // *T_12_* //… … // *T_1m1_*}, then HEROS is a typical real-time multi-tasking system like SDREAM. When *j* = *1, H* = {*T_11_*→*T_21_*→… …→*T_n1_*}, HEROS is thus a typical event-driven system like TinyOS. Figure 3 shows the schematic diagram of HEROS.

Furthermore, based on the fault-tolerant component-based hardware architecture of Mobi+ cards, the fault-tolerant mechanism and resource awareness techniques are thus adopted:

- *Resource awareness policies:* HEROS employs the *meta* language (kernel modeling language, KML) to model system architecture and to abstract system actions so as to improve the efficiency of system primitives. Moreover, HEROS implements a micro *meta* file system-LiveFile [14] that adopts the Writing Rotation Process (WRP) technique to improve program density and then decrease memory consumption.
- *Fault-tolerant mechanism:* In view of the multi-microcontroller multi-transceiver hardware architecture, HEROS implements a self-adaptive task-migration mechanism based upon the MCUs heartbeat detection module and the LINDA-based parallel communication mechanism. When an abnormal event is detected by the heartbeat logics, the tasks (*events* and *threads*) in the fault microcontroller can be immigrated into the back-up microcontroller.

#### B. Resource Awareness Wireless Protocol

A reliable wireless communication mechanism between buses and stations in the complex urban environment is one of the key challenges for the Mobi+ system. In order to improve the connection reliability and at the same time reduce the energy consumption, one solution is to decrease network traffic. In the Mobi+ system, the *meta* data with the code-book based data compression techniques are adopted to minimize packet size, and thus to optimize bandwidth use and increase communication reliability. Moreover, the hamming code technique is used to correct wireless communication errors.

**Figure 4.**
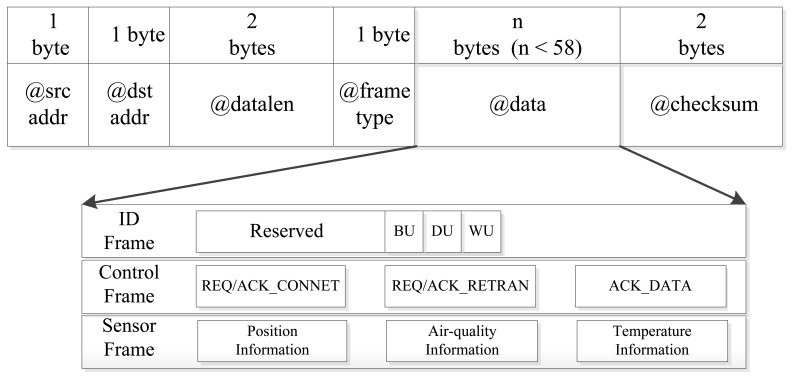
Data format of Mobi+ frame.

The Mobi+ frame structure is defined as follows (see Figure 4): @*srcaddr* (1 bytes) + @*dstaddr* (1 byte) + @*datalen* (2 bytes) + @*datatype* (1 bytes) + @*data* (*n* bytes) + @*checksum* (2 bytes). The @*srcaddr* and @*dstaddr* fields fill in the addresses of source and destination nodes, *i.e.*, the address identifier of the Mobi+ (bus and station) cards which are configured during system initialization. The Mobi+ system supports two kinds of wireless connection: unicast and broadcast. In broadcast mode, the value of @*dstaddr* equals zero. The value of @*datalen* indicates the total field length of @*datatype*, @*data* and @*checksum*, which is between 1 and 58 bytes. The @*checksum* field stores Cyclic Redundant Check (CRC) code.

The Mobi+ system supports three frame types: *Control* frame, *ID* frame and *Sensor* frame, defined in @*datatype* field. The *ID* frame (1 byte) indicates the classes of the DWB passengers defined as follows: bit0 represents wheelchair user (WU), bit1 represents disabled user (DU), bit2 represents blind user (BU), and bit3∼7 are reserved; the *Sensor* frames are used to hold the sampling data from sensors on bus, which stores the following information: the position data from GPS, and the environmental data from air-quality sensors and temperature sensor; the *Control* frames have five types (1 byte): REQ_CONNET, ACK_CONNECT, REQ_RETRAN, ACK_RETRAN, and ACK_DATA, which are responsible for the control commands of connect request/acknowledge, retransmission request/acknowledge and data acknowledge, correspondingly.

## 4. Description of Mobi+ System

The Mobi+ system, as an effective complement to the urban transportation system, aims to provide accessibility of urban public transportation to people with disabilities. Three main ICT function are provided by the Mobi+ system, *i.e.*, DWB detection & Alarm notification function, BUS position & environmental monitoring function and BUS-Station wireless communication function. Thus the accessibility services are achieved in the following subsystems: wireless subsystem, BUS subsystem and station subsystem, respectively.

### 4.1. Wireless Subsystem

The wireless subsystem, which is responsible for data exchange and network connection between buses and stations, is composed of two functional modules: the bus communication module and the station communication module. The core of communication module is the multi-MCU multi-transceiver Mobi+ card that adopts the fault-tolerant component-based hardware architecture (shown in Figure 2).

**Figure 5.**
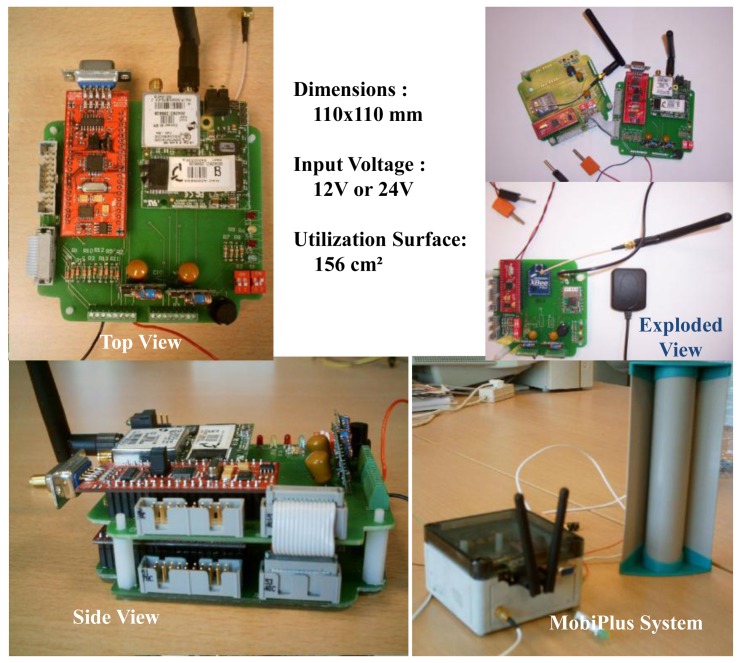
Prototype of the Mobi+ card.

In Figure 5, the physical prototype of Mobi+ card is presented, which includes two functional boards: the top board is the master, equipped with an ARM7TDMI-based microcontroller (NXP LPC2106) with WiFi and RFID (Station)/GPS(Bus); the bottom one is the slave, equipped with the second ARM7TDMI-based microcontroller (NXP LPC2106) with ZigBee and other functional modules, e.g., gas sensors (Bus)/alarms (Station), *etc.* In the Mobi+ card, WiFi and ZigBee wireless media are installed and work simultaneously. The directional antennas are also installed for WiFi and ZigBee connections, respectively.

To ensure a reliable data exchange in the complex urban wireless environment, our solution is the multi-microcontroller multi-transceiver hardware architecture (as discussed in Section 2.1), and the resource awareness software architecture (as discussed in Section 2.2). The hardware architecture enables the synchronous data transmission by the redundancy implementation of wireless connections via two different wireless links; and the resource awareness wireless protocol provides acknowledge and retransmission mechanism to ensure a reliable data transmission.

### 4.2. Bus Subsystem

**Figure 6.**
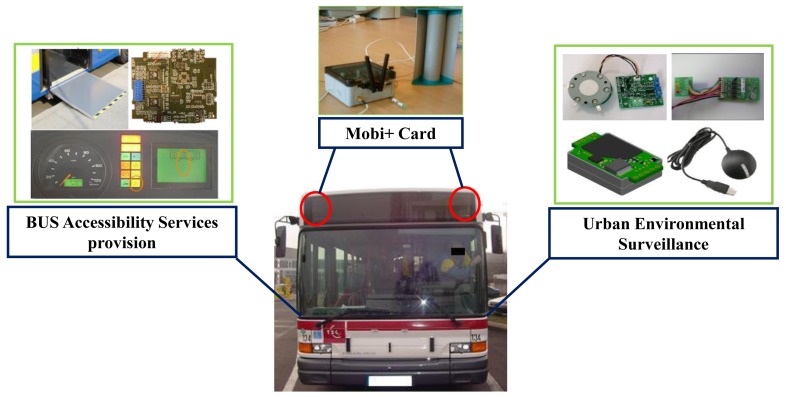
BUS subsystem of Mobi+.

The bus subsystem contains two main functional modules: the environmental surveillance module and the bus accessibility service provision module. Figure 6 shows the schematic diagram of Mobi+ bus subsystem.

The environmental surveillance module collects the urban air-quality data along the bus line at different times of the day when buses run in the city. Through the statistical analysis of the collected environmental data, the Mobi+ back-end system will release the on-line urban environmental quality reports and then will provide the surveillance service of city environmental quality in real-time. The Mobi+ system acquires the bus position and time information via GPS receiver and then collects the urban environmental data periodically via different environmental sensors. The environmental data are firstly stored into the memory store unit in the Mobi+ bus card and then are sent to the Mobi+ station subsystem when the bus is approaching a station and the wireless connection between the bus and the station is established.

The bus accessibility service provision module provides a dedicated bus parking & access service for the DWB passenger according to the DWB type which is recognized by the station subsystem (Section 3.3) and then is transmitted to the BUS through the wireless communication subsystem (Section 3.1). When a DWB type message is received in the BUS peer, the alarm actions are driven to inform the BUS driver the DWB passenger information at the next bus stop (station) including the light blinking frequency of the luminous indicator and the DWB type of voice message announcement. If the DWB type is WU, the BUS will automatically deploy a pallet (wheelchair access platform) when stopping at the station. This access platform provides a cross-bridge over the pavement and the bus entrance for the DWB passengers so the WU can autonomously access the BUS. If the DWB type is BU, the acknowledge frame will be sent back to the station peer, and a specific sound will ring to inform BU of the bus arrival at the station.

**Figure 7.**
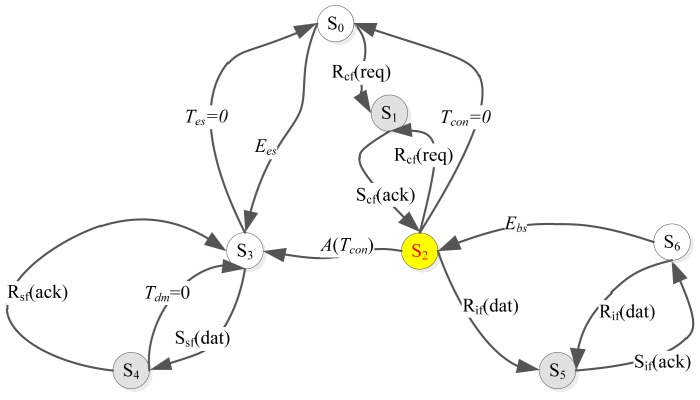
State-Transition Diagram of BUS subsystem.

The Mobi+ system is an event-driven state-transition system. Given the definitions of Mobi+ system behaviors in Table 1, the operation states of the bus subsystem are described as follows: *S0* is the initialization state where the system performs the periodic sampling tasks to collect environmental data and also to read the connection request; *S2* (*S3*) is the network connection state where the network connection between the bus and the station is established by exchanging the Mobi+ control frames. In this state, the bus sends the sampling data to the station and then receives the DWB type information from the station; *S6* is the service provision state in which the bus provides different bus parking & access services according to the DWB type. *S1, S4*, and *S5* are the system intermediate states which indicate the state transition conditions and the relative event-driven mode of Mobi+ bus subsystem, shown in Figure 7.

### 4.3. Station Subsystem

**Figure 8.**
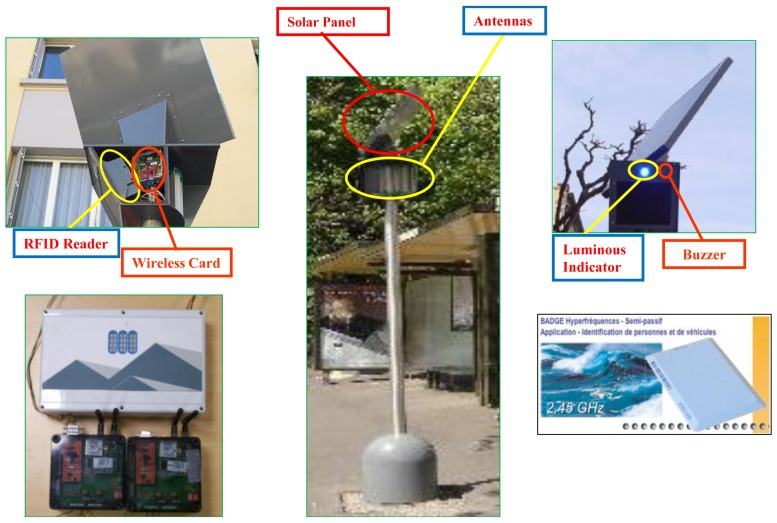
Station subsystem of Mobi+.

The station subsystem consists of two main functional modules: the DWB detection module and the bus arrival notification module. Figure 8 shows the schematic diagram of the Mobi+ station subsystem.

Based on RFID techniques, the DWB detection module recognizes three DWB types: wheelchair users, disabled users and blind users. The DWB passengers will take a specific urban public transportation ticket which is a RFID card (tag) where the disabled type and the identity of the people with disabilities are registered. When a DWB passenger is waiting at a bus station, the people with disabilities will be detected and recognized by the RFID reader equipped on the communication pillar at the bus station. In Mobi+, the RFID reader adopts the model of PRG 55 Siemens 10, which can detect the RFID tags within the range of 10 meters in the urban environment. The RFID data will be sent directly to the station card via the UART serial port, and then be further processed and encapsulated into an ID frame that will be sent to a bus via two possible wireless connections when the bus is approaching and the network communication is established.

The arrival notification service module provides a dedicated notification service of bus arrivals for the DWB passengers. The luminous indicator will be lighted when the RFID reader detects a DU or WU tag, and then begin to flick when the bus is approaching. The buzzer will begin to ring at low-frequency when detecting a BU tag, and then the buzzer ringing frequencies are gradually increased to signal an incoming bus. The luminous indicator and the buzzer will stop operating when the bus leaves the station and the network connection is disconnected.

**Figure 9.**
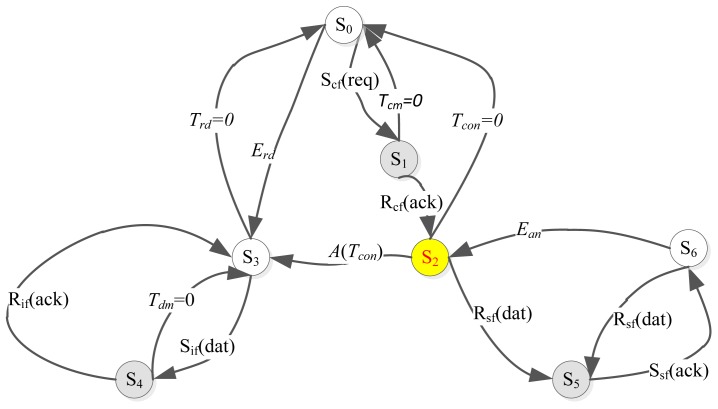
State-transition diagram of station subsystem.

The operation states of the station subsystem are described as follows: *S0* is the initialization state in which the system performs the periodic DWB detection & recognition task and also the periodic connection requests sending task; *S2(S3)* is the network connection state where the station sends the DWB class data to the bus and receives the sensor sampling data from the bus; *S6* is the arrival notification state where the station provides a dedicated notification service to signal an incoming bus for different DWB classes. *S1, S4*, and *S5* are the system intermediate states which indicate the state transition conditions and the relative event-driven mode of Mobi+ station subsystem, as shown in Figure 9.

## 5. Conclusions

In this paper, from the perspectives of the system architecture and components, we introduce the Mobi+ system, which aims to facilitate access to the urban public transportation system for people with disabilities (*i.e.*, disabled, wheelchair and blind passengers), and also to collect urban environmental data along the bus lines at different times of the day.

**Figure 10.**
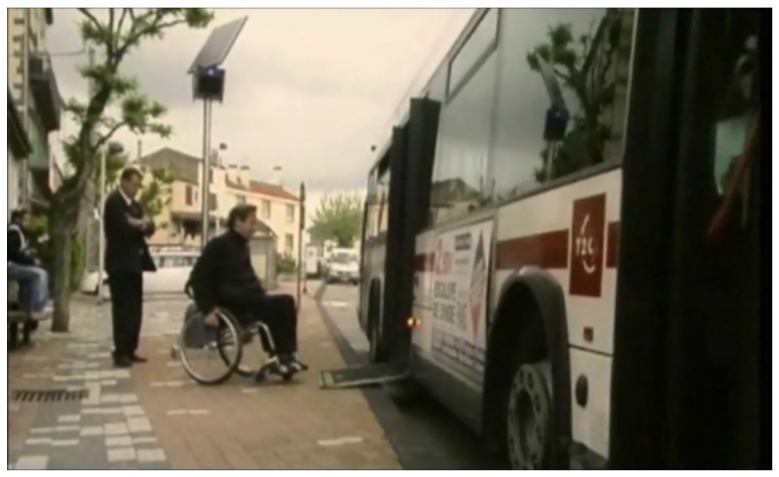
Mobi+ prototype system at Clermont-Ferrand, France.

The Mobi+ card is a dedicated low-cost embedded system which integrates a fault-tolerant component-based hardware architecture with a fault-tolerant resource awareness software system that combines a real-time hybrid embedded operating system micro-kernel and a dedicated wireless application protocol. The Mobi+ system is an event-driven state-transition system that can provide the DWB detection/alarm notification services in the station peer, the environmental surveillance/bus parking & access services in the bus peer, and the wireless communication service between buses and states in the wireless subsystem. Corresponding with the multi-microcontroller multi-transceiver hardware architecture, a fault-tolerant resource awareness communication and scheduling mechanism has been implemented in the Mobi+ system to ensure the reliability of data exchange and service provision. At present, the Mobi+ system has been implemented on the buses and stations of line‘2’ in the city of Clermont-Ferrand (France), as shown in Figure 10.

Experiential results show that the performance of Mobi+ prototype system meets the design expectations and can provide an effective bus access service for people with disabilities by minimizing significantly the total bus route time. Moreover, thanks to its small form factor and low energy consumption, the Mobi+ system is deployed easily in the buses and bus stations (powered by batteries with solar panels). In the future, the Mobi+ system will be applied to more city bus lines so as to build an urban environmental surveillance network that extends the auxiliary bus parking & access service to passengers with a wider variety of disability types. To achieve these objectives, the Mobi+ system should have the capabilities of inter-vehicle communication (IVC) and Internet access (IA), so that it can provide smart urban transportation services, including transportation condition reporting (traffics, accidences …), urban environment data collection (air quality, temperature …), Internet access, *etc.*

## Figures and Tables

**Table 1.** Definitions of Mobi+ system.

**Types**	**Symbols**	**Descriptions**
Services	*E_es_*	Environmental surveillance task
	*E_bs_*	Bus accessibility service provision task
	*E_rd_*	DWB detection and recognition task
	*E_an_*	Bus arrival notification task
Frames X_frame_(Data)	*X:*= *R/S*	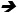 *receive/send* operation type
	*FRAME:*= *cf/if/sf*	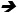 *control/ID/sensor* frame type
	*DATA:*= *req/ack/dat/con*	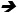 *request/acknowledge/data/connection frame data*
Timers	*T_cm_*	Latency time of control message acknowledge
	*T_dm_*	Latency time of data message (ID/sensors) acknowledge
	*T_con_*	Period of wireless connection
	*T_es_*	Period of *E_es_* task
	*T_rd_*	Period of *E_rd_* task
